# Enhancing Mechanical Properties of Graphene/Aluminum Nanocomposites via Microstructure Design Using Molecular Dynamics Simulations

**DOI:** 10.3390/ma17184552

**Published:** 2024-09-16

**Authors:** Zhonglei Ma, Hongding Wang, Yanlong Zhao, Zhengning Li, Hong Liu, Yizhao Yang, Zigeng Zhao

**Affiliations:** 1 School of Mechanical Engineering, Lanzhou Jiaotong University, Lanzhou 730070, China; mazl1205@163.com (Z.M.); ylz0109xian@163.com (Y.Z.); liuh@lzjtu.edu.cn (H.L.); yangyizhao0812@163.com (Y.Y.); zhaozigeng2022@163.com (Z.Z.); 2 School of Materials Science and Engineering, Lanzhou Jiaotong University, Lanzhou 730070, China; lzn015@mail.lzjtu.cn

**Keywords:** brick-and-mortar structure, graphene/aluminum nanocomposites, nanoindentation, high-entropy alloy coating, dislocation

## Abstract

This study explores the mechanical properties of graphene/aluminum (Gr/Al) nanocomposites through nanoindentation testing performed via molecular dynamics simulations in a large-scale atomic/molecular massively parallel simulator (LAMMPS). The simulation model was initially subjected to energy minimization at 300 K, followed by relaxation for 50 ps under the NPT ensemble, wherein the number of atoms (N), simulation temperature (T), and pressure (P) were conserved. After the model was fully relaxed, loading and unloading simulations were performed. This study focused on the effects of the Gr arrangement with a brick-and-mortar structure and incorporation of high-entropy alloy (HEA) coatings on mechanical properties. The findings revealed that Gr sheets (GSs) significantly impeded dislocation propagation, preventing the dislocation network from penetrating the Gr layer within the plastic zone. However, interactions between dislocations and GSs in the Gr/Al nanocomposites resulted in reduced hardness compared with that of pure aluminum. After modifying the arrangement of GSs and introducing HEA (FeNiCrCoAl) coatings, the elastic modulus and hardness of the Gr/Al nanocomposites were 83 and 9.5 GPa, respectively, representing increases of 21.5% and 17.3% compared with those of pure aluminum. This study demonstrates that vertically oriented GSs in combination with HEA coatings at a mass fraction of 3.4% significantly enhance the mechanical properties of the Gr/Al nanocomposites.

## 1. Introduction

Aluminum alloy exhibits low density, high strength, high ductility, high corrosion resistance, and good processability. Graphene (Gr), a single-atom-thick and two-dimensional carbon sheet, has attracted the attention of researchers worldwide because of its outstanding mechanical, electrical, and thermal properties [1,2]. Gr sheets (GSs) are considered ideal reinforcing materials for polymers, metals, and ceramics. Consequently, graphene/aluminum (Gr/Al) nanocomposites have become the focal point in metal matrix composite research [3,4]. The incorporation of Gr with a high elastic modulus (1 ± 0.1 TPa) and high tensile strength (130 ± 10 GPa) [5] in Al is an effective method of improving its mechanical properties [6]. Gr/Al composites have superior mechanical performance and functional characteristics compared to nonreinforced materials. For example, Wang et al. [7] prepared Gr-reinforced Gr/Al nanocomposites using a flake powder metallurgy method and discovered that the addition of only 0.3 wt% Gr to the Gr/Al nanocomposites increased the yield strength by 62%. Similarly, Shin et al. [8] discovered that mechanical ball milling and hot rolling led to a twofold increase in the tensile strength of single-layer, Gr-reinforced Gr/Al nanocomposites compared to monolithic aluminum. Yan et al. [9] prepared Gr/Al composites using powder metallurgy. They found that the addition of 0.5% mass fraction of Gr increased the yield strength of the composites by approximately 50% while maintaining plasticity. In addition, Wang et al. [10] prepared Al_2_O_3_-decorated, reduced Gr oxide (RGO)-reinforced Al6061 composites. The results showed that by adding only 0.1 wt% of Al_2_O_3_/RGO, the yield and tensile strengths were increased by 49% and 43%, respectively, compared with those of pure Al6061 prepared through the same process.

Although numerous experiments have demonstrated that Gr can improve the mechanical performance of metal matrix nanocomposites, their mechanical properties are governed by various interatomic interactions. Consequently, although experimental methods can reveal the macromechanical enhancements in mechanical properties, they do not fully elucidate the strengthening mechanisms from a microscopic perspective or interactions between different atoms. Therefore, many researchers have initiated numerical simulation studies on Gr-reinforced metal matrix nanocomposites. Molecular dynamics (MD) simulation is an effective numerical method that can elucidate the atomic-scale deformation mechanisms of materials under different loading conditions, avoiding the need for intricate experimental conditions. Consequently, MD simulation has become a pervasively used technique across different scientific research domains [11,12]. For example, Zhu et al. [13] investigated the effects of different Al crystal orientations and Gr layer spacings on the strengthening mechanism of Gr/Al matrix composites under uniaxial compression. Chang et al. [14] simulated the mechanical behavior of Gr/Ni matrix composites under nanoindentation loading and highlighted that the hardness of the composites was dependent on the number of Gr layers present. Lei et al. [15] performed a nanoindentation simulation study on Gr/Al matrix composites. The simulation results showed that the load-bearing capacity of the composites containing Gr was 4–5 times stronger than that of the pure Al matrix. Han et al. [16] investigated the deformation behavior and mechanical properties of Gr/Al nanocomposites with tilted GSs under nanoindentation testing. Muller et al. [17] investigated the failure mechanisms of Gr/Ni nanocomposites under different loading orientations using MD simulations, where cracks were formed in the Ni matrix, which led to failure mechanisms such as dislocation nucleation, Gr bond breakage, and delamination. Li et al. [18] investigated the mechanical properties and microscopic deformation mechanisms of Gr/nanotwinned Al matrix composites under uniaxial tensile loading using MD simulations. Their findings showed that the synergistic effect of twin boundaries (TBs) and Gr led to a linear increase in the elastic modulus, strength, and toughness of the composites with increasing number of Gr layers.

The orientation and arrangement of Gr during processing significantly affect material mechanical properties. Changing the orientation and arrangement of Gr in the matrix effectively improves the mechanical properties of nanocomposites. An innovative way of Gr incorporation into nanocomposites is the preparation of the brick-and-mortar structure. This structure is inspired by natural biological structures, such as the abalone shell. The microstructure of the abalone shell is similar to the brick-and-mortar structure used in architectural design, with hard minerals forming the bricks and soft organic matter composing the mortar [19,20]. Similarly, high-strength Gr can serve as the “brick” in natural materials, while metals can function as the “mortar”. Huang investigated the effect of Gr with a brick-and-mortar structure on the mechanical properties of Gr/Ni nanocomposites through nanoindentation testing simulations. They revealed that the strength and hardness of the nanocomposites decreased as the length of the Gr layers increased [21].

At present, most MD simulation studies on Gr-reinforced metal matrix nanocomposites with a brick-and-mortar structure have focused on nickel-based materials, with limited research being conducted on Al-alloy matrix nanocomposites. In addition, existing studies have demonstrated that although the brick-and-mortar structure can significantly improve the elastic modulus of Gr/Al nanocomposites, it reduces their hardness. Therefore, it is necessary to investigate the influence of the arrangement and coating of Gr with the brick-and-mortar structure on the mechanical properties of Gr/Al nanocomposites.

To alleviate the weakening effect of the brick-and-mortar structure on material hardness while increasing the elastic modulus and to obtain Gr/Al nanocomposites with significantly improved elastic modulus and hardness compared to pure aluminum, it may be reasonable to coat Gr with a layer of high-entropy alloys (HEAs). Unlike conventional alloys, which typically consist of one or two primary elements, HEAs comprise five or more key elements in equal or nearly equal atomic proportions [22,23]. HEA coatings on Gr surfaces exhibit multiplicity mixing, which inhibits atoms from achieving a regular structural arrangement [24]. This leads to a more disordered atomic configuration. In this study, MD simulations were employed to construct models of Gr/Al nanocomposites with and without HEA coatings by inserting GSs into the aluminum matrix with horizontal and vertical placement. We compared the dislocation evolution processes and analyzed the variations in the indentation force, hardness, and elastic modulus during the nanoindentation testing of the various models. Furthermore, we examined in detail the mechanism underlying interactions between the dislocation network generated by indentation and the GSs. The results indicated that the HEA (FeNiCrCoAl) coatings significantly enhanced the hardness of the Gr/Al nanocomposites. The mechanical properties of the vertically aligned GSs were particularly pronounced, resulting in increased hardness values and elastic modulus of the nanocomposites by 17.3% and 21.5%, respectively, compared with those of pure aluminum. This study is expected to guide the design of nanocomposite materials with specific mechanical properties.

## 2. Materials and Methods

**Figure 1:**
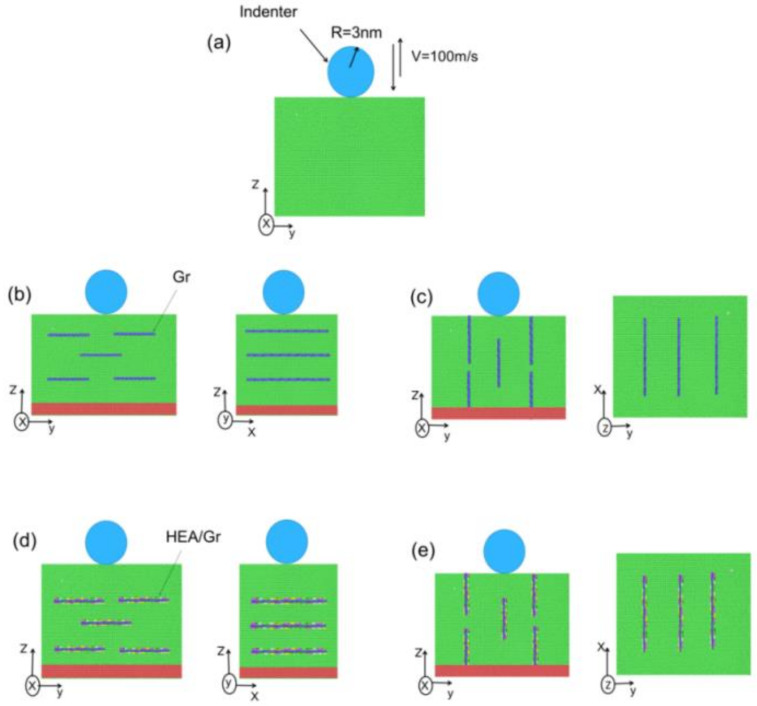
Schematic of the nanoindentation models: pure aluminum (**a**), Gr/Al-level3 (**b**), Gr/Al-vertical3 (**c**), HEA/Gr/Al-level3 (**d**), and HEA/Gr/Al-vertical3 (**e**).

MD simulations were performed using the large-scale simulator LAMMPS. To simulate the nanoindentation of Gr/Al nanocomposites, the first step was to construct the nanoindentation model. Five models were constructed, as shown in Figure 1a–e: Figure 1a shows a single-crystal aluminum model without Gr containing 329,940 aluminum atoms. Figure 1b,c show five defect-free GSs embedded into the aluminum matrix along the horizontal and vertical directions, respectively, after arranging them in a sheet structure. Figure 1d,e show the HEA (FeNiCrCoAl) coatings added to the GSs based on the models in Figure 1b,c. The size of the monolayer GS in all Gr/Al nanocomposite models was 96 Å × 70 Å, the number of carbon atoms was 12,753, and the mass fraction of Gr was 1.75%. The mass fraction of HEA in the models with added HEA coatings was 3.4% in all models. For descriptive convenience, the five models were defined as pure aluminum, Gr/Al-level3, Gr/Al-vertical3, HEA/Gr/Al-level3, and HEA/Gr/Al-vertical3. The Gr horizontally aligned model has Gr along the y- and x-directions for the armchair and zigzag directions, respectively, and the Gr vertically aligned model has Gr along the z- and x-directions for the armchair and zigzag directions, respectively.

As shown in the schematic of the nanoindentation model in Figure 1, the blue sphere represents a virtual indenter. Because a virtual indenter is used for nanoindentation, the indenter shown in Figure 1 only represents the indenter size with a radius of 3 nm, and the initial gap between the indenter and the upper surface of the substrate is 0.5 nm. To prevent the movement of the workpiece during the indentation process, the atoms at the bottom of the 5 Å were set as a fixed layer, and the workpiece dimensions were 220 × 192 × 131 Å. During the construction of the Gr/Al nanocomposite model, variations in the orientation of the embedded Gr resulted in slight discrepancies in the number of atoms removed during the extraction of aluminum atoms. However, the effect of these differences on the overall volume change of the models was negligible. Therefore, the indentation force per unit volume of the different models is not addressed in this study. The simulation employs periodic boundary conditions in the x- and y-directions, whereas free boundary conditions are applied in the z-direction.

Before initiating the nanoindentation process, each model was subjected to energy minimization, followed by a relaxation period of 50 ps to ensure full equilibrium of the system. Throughout the indentation process, the atomic number (N), pressure (P), and temperature (T) were kept constant, with the temperature set at 300 K. The NPT system was selected for relaxation. Nanoindentation was performed through a displacement-controlled loading process with a predetermined indenter velocity and maximum indentation depth. The indenter velocity was set at 1 Å/ps (100 m/s) for the nanoindentation simulation, with a maximum indentation depth of 3 nm. Subsequently, the indenter was unloaded at the same speed upon reaching the maximum depth. The indentation force was recorded at each simulation time step during both the indentation and unloading phases. The simulation time step was set to 1 fs. The mathematical expression representing the force applied by the indenter tip on the workpiece atoms during the indentation process is as follows:

*F*(*r*) = −*K*(*r* − *R*)^2^, (1)

where *F*(*r*) denotes the repulsive force between the indenter tip and an atom at a distance of *r*; *K* represents the force constant; *R* represents the indenter radius.

Accurate selection of a potential function is important for ensuring the precision and reliability of MD simulation results. In this study, the combined potential approach was adopted, where the interaction among aluminum atoms was elucidated using the embedded atom potential proposed by Mishin et al. [25]. The interaction among carbon atoms was described using the AIREBO potential [26], and the interaction among metal atoms was characterized using the embedded atom potential introduced by Farkas et al. [27] within the HEA model. In addition, the interaction between carbon and metal atoms was described using the Lennard-Jones (L-J) potential:

(2) $$E_{ij}=4\varepsilon_{ij}[(\frac{\sigma_{ij}}{r_{ij}}{)}^{12}-\;(\frac{\sigma_{ij}}{r_{ij}}{)}^{6}],$$

where *E_ij_* denotes the potential energy between the ith and jth atoms, *r_ij_* denotes the distance between two atoms, *ε_ij_* denotes the potential depth, and *σ_ij_* denotes the equilibrium interatomic distance. The values of these parameters are listed in Table 1. The L-J potential has been widely used to study the mechanical properties and deformation mechanisms of Gr/Al nanocomposites [3,28]. In this study, we used the Open Visualization Tool (OVITO) [29] to analyze the evolution of microstructure during the indentation process, common neighbor analysis [30] to analyze the local atomic crystal structure, and the dislocation extraction algorithm (DXA) [31] to classify the types of dislocations.

## 3. Results and Discussion

### 3.1. Dislocation Evolution

**Figure 2:**
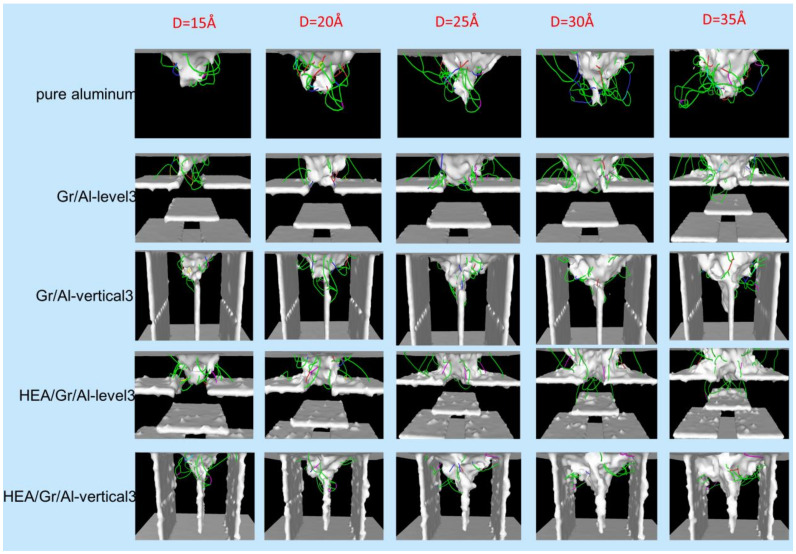
Dislocation distributions observed along the x-axis for the five models, namely pure aluminum, Gr/Al-level3, Gr/Al-vertical3, HEA/Gr/Al-level3, and HEA/Gr/Al-vertical3, at indenter displacements of d = 15, 20, 25, 30, and 35 Å. Dislocations are colored according to their Burgers vector. Green: 1/6<112>; Dark blue: 1/2<110>; Pink: 1/6<110>; Yellow: 1/3<100>; Bright blue: 1/3<111>; Red: others.

As shown in Figure 2, the dislocation formation processes and mechanisms in the five models were investigated. The total number of dislocations exhibited an overall increasing trend for all the models. The number of dislocations peaked when the indenter reached a maximum depth of d = 35 Å.

In pure aluminum, the generation mechanism of dislocations was similar to the mechanism observed in other face-centered cubic (FCC) crystals, as reported in numerous simulation studies [32,33,34,35]. Prismatic dislocation rings were emitted from pits along the slip direction, and dislocations had a high degree of mobility due to the absence of obstacles that could obstruct them. Dislocations were surrounded by pits, forming a dense network at that location.

In the Gr/Al-level3 model, the dislocation formation mechanism during the initial stage resembled that observed in pure aluminum. Dislocations were initiated around the crater and propagated downward. They reached the surface of the first Gr layer when the indenter displacement was 12.2 Å. Subsequently, because of the hindering effect of GSs, the dislocations propagated laterally above the first Gr layer, leading to the generation of additional dislocation rings. When the indentation force exceeded a specific threshold, the propagation of Shockley dislocations occurred in the downward direction. However, the number of dislocations that reached the upper surface of the second Gr layer is limited.

In the HEA/Gr/Al-level3 model, the generation and downward extension of dislocations were similar to those observed in the Gr/Al-level3 model; however, dislocations started to appear on the upper surface of Gr coated with HEAs at an indenter displacement of 11.1 Å. Notably, dislocations did not propagate downward from the pit, indicating that HEA-coated Gr demonstrated enhanced load-bearing capacity and more pronounced resistance to the indentation force. This characteristic contributed to the enhancement of nanocomposite hardness. In the HEA/Gr/Al-level3 model, dislocations propagated downward through the gap in the middle of the first Gr layer earlier than in the Gr/Al-level3 model, resulting in many dislocations reaching the surface of the second Gr layer at the maximum indenter displacement. Almost no dislocation loops were formed because dislocations were more strongly impeded and absorbed by Gr after the incorporation of the HEA coating; thus, the dislocation lines were generally shorter.

In the Gr/Al-vertical3 model, dislocations began to nucleate and grow downward under the indentation force. They continued to extend along the horizontal and vertical directions after contacting the intermediate GSs until they began to decrease in number when the displacement reached 20 Å. This result indicated that GSs played a crucial role in enhancing the mechanical properties of the nanocomposites. Subsequently, the number of dislocations increased to a level close to that before the reduction. Finally, the dislocations failed to nucleate on the two outer sides of the GSs. In the HEA/Gr/Al-vertical3 model, the dislocation nucleation tendency was similar to that observed in the Gr/Al-vertical3 model due to the similarity in the arrangement of GSs. The difference lay in the fact that dislocations reached the top of the middle GS earlier than in the Gr/Al-vertical3 model. Subsequently, the tendency of the dislocations to extend horizontally became more pronounced. This indicated that the GSs have a stronger hindering effect on the dislocations after HEA coating. In addition, the number of dislocations was significantly higher in the HEA/Gr/Al-vertical3 model than in the Gr/Al-vertical3 model, suggesting that the coating enhanced the mechanical properties of the nanocomposites.

### 3.2. Crystal Structure and Stacking-Fault (SF) Evolution

**Figure 3:**
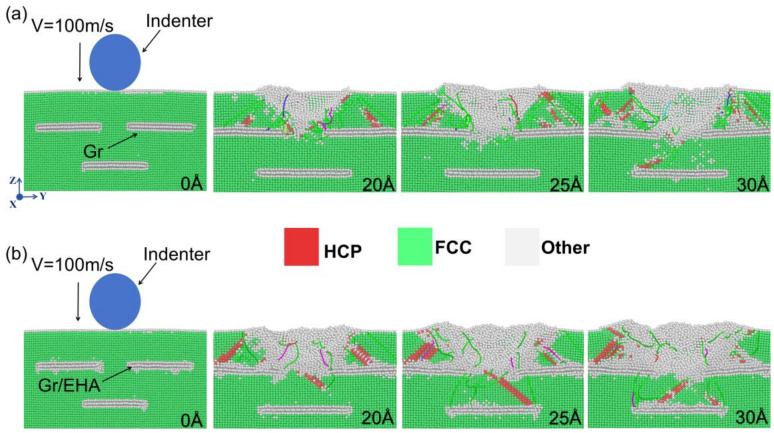
Side views of the y–z-planes of Gr/Al-level3 (**a**) and HEA/Gr/Al-level3 (**b**) at indentation depths of 0, 20, 25, and 30 Å. The viewing direction is along the x-axis.

**Figure 4:**
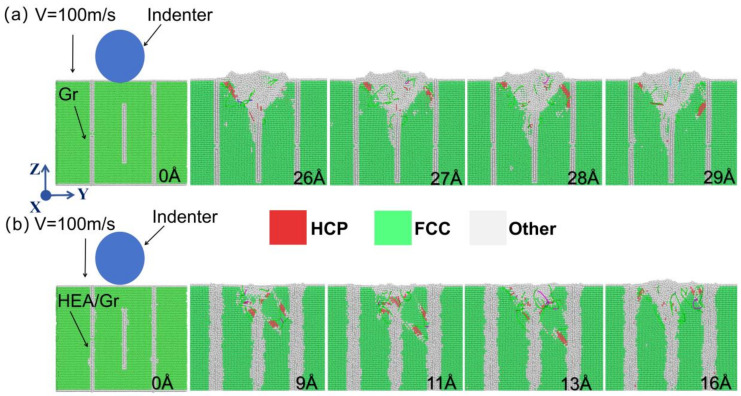
Side views of the y–z-planes of Gr/Al-vertical3 (**a**) at indentation depths of 0, 26, 27, 28, and 29 Å and HEA/Gr/Al-vertical3 (**b**) at indentation depths of 0, 9, 11, 13, and 16 Å. The viewing direction is along the x-axis.

The effects of HEA coatings on the crystal structure and SF motion in the Gr/Al nanocomposites with a horizontal brick-and-mortar structure are illustrated in Figure 3. In FCC crystals, the SF region is bounded by partial dislocations and consists of two planes of atoms with a hexagonal close-packed (HCP) structure [36,37]. As the indenter moves downward, an HCP structure is formed, generating an SF that moves downward. As shown in Figure 3a, when the SF is obstructed by the GSs, it deflects to the left and moves upward, similar to the phenomenon observed in Figure 4a. However, in the presence of HEA coatings on the Gr surface, the reversed SF movement is not observed because the HEA atoms are arranged in a heterogeneous manner and can absorb the SF, as depicted in Figure 3b and Figure 4b.

**Figure 5:**
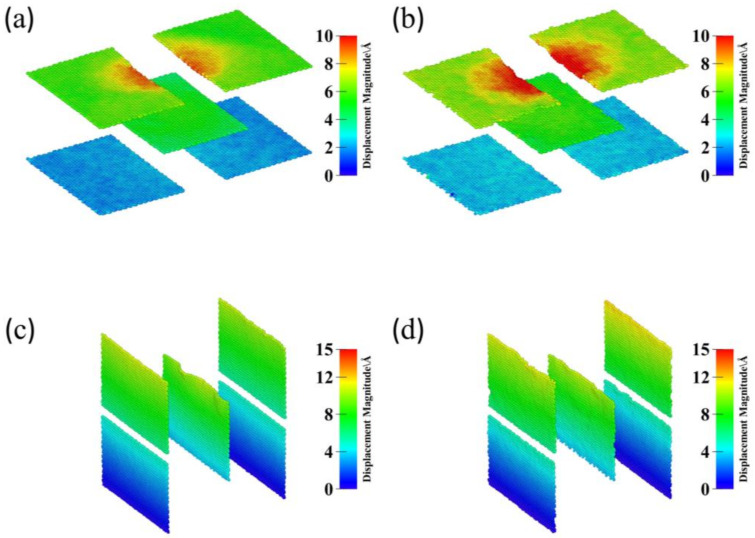
In-plane height profiles of Gr at an indentation depth of 30 Å for models Gr/Al-level3 (**a**), HEA/Gr/Al-level3 (**b**), Gr/Al-vertical3 (**c**), and HEA/Gr/Al-vertical3 (**d**).

At an indenter displacement of 25 Å, the first Gr layer in the Gr/Al-level3 model remained horizontal, while the HEA-coated Gr layers were bent. This bending may be because the HEA coating absorbed some of the dislocations, which induced deformation of the Al matrix and GSs. This bending allowed more dislocations to move toward the second Gr layer, resulting in improved mechanical properties. Figure 5 shows the in-plane height profiles of GSs at an indentation depth of 35 Å for models Gr/Al-level3, HEA/Gr/Al-level3, Gr/Al-vertical3, and HEA/Gr/Al-vertical3, as described by the displacement vector method. This method uses the magnitude of relative displacements for color coding. The GSs with HEA coatings exhibited a larger displacement at 35 Å, which improved the participation of the Al matrix in the deformation process. These findings demonstrate that during the nanoindentation testing of the Gr/Al nanocomposites, dislocations were obstructed by GSs as the indentation depth increased, thereby affecting the slip process. The HEA coating enhanced the flexibility of GSs and altered the expansion tendency of the plastic zone.

### 3.3. Total Dislocation Length and Loading Indentation Force Analysis

**Figure 6:**
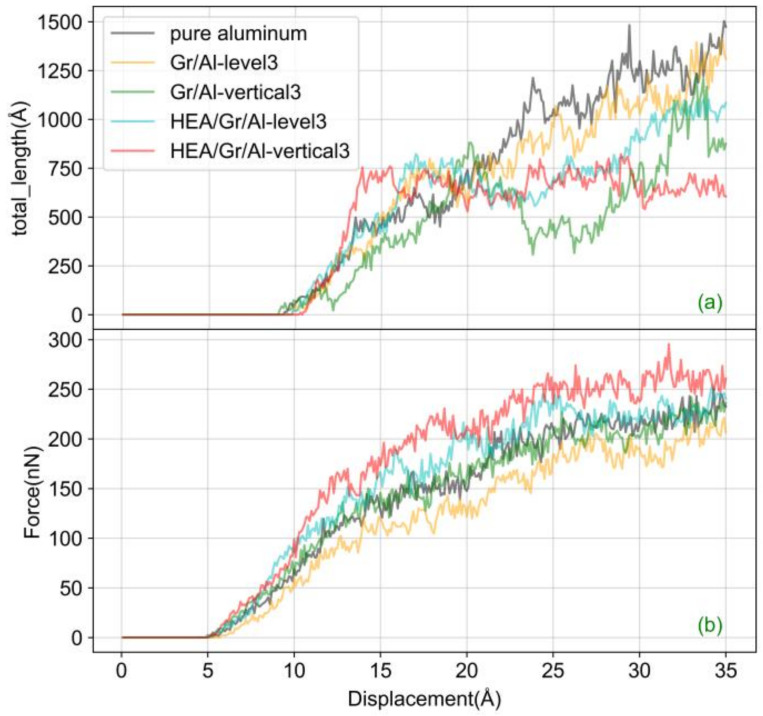
Evolution of total dislocation length (**a**) and indentation force (**b**) with indenter displacement.

Figure 6a illustrates the evolution and relationship between the total dislocation length and indenter displacement. The dislocations begin to sustain nucleation between d = 9.0 and 9.9 Å in the five models. In the pure aluminum model, the length of the dislocation network increases steadily as the indenter displacement increases. The fluctuation in the total dislocation length is caused by atomic natural dislocations, and the interaction between the dislocations slightly decreases the dislocation length.

In the Gr/Al-level3 curve, the total dislocation count decreases twice at d = 14 and 18 Å. The reduction in dislocations at d = 14 Å is relatively slight owing to the dislocations reaching the upper Gr surface, where Gr begins to impede dislocation growth. However, dislocations initially grow from the upper region of the gap between the two Gr layers, leaving room for dislocation nucleation in the upper part of Gr. At this stage, the dislocation hindrance induced by Gr is minimal, resulting in minimal fluctuations in the dislocation count curve. The number of dislocations continues to increase transversely above the first Gr layer, and a significant decrease in the number of dislocations occurs until d = 18 Å. Accompanied by significant fluctuations, a linear increase in the number of dislocations is observed.

In the HEA/Gr/Al-level3 model, the pattern of the dislocation curves is roughly similar to that observed in the Gr/Al-level3 model, implying that the variation in the number of dislocations is primarily influenced by the Gr arrangement. However, a notable distinction is observed in the HEA/Gr/Al-level3 curves. The point of inflection in the number of dislocations occurs earlier than that observed in the Gr/Al-level3 curves and is significantly lower than that observed in the Gr/Al-level3 model within the range of d = 20–35 Å. This difference can be attributed to the crucial role played by Gr in impeding dislocations at this stage, and the presence of coated Gr provides a more pronounced hindering effect on dislocations, which positively influences the mechanical properties of the Gr/Al nanocomposites.

In the Gr/Al-vertical3 model, the initial ample space for dislocation propagation leads to a rapid increase in the dislocation curve until 20 Å. Subsequently, the curve experiences a temporary decline as dislocations encounter hindrance from the top of the intermediate GS. Beyond 20 Å, both lateral and vertical dislocation extensions are impeded, resulting in a sharp decrease in the number of dislocations. Next, the interactions between different dislocations lead to fluctuations in the total dislocation count within a certain range. After 30 Å, the dislocations continue to increase downward, resulting in a continuous increase in the dislocation count until reaching the maximum value.

The HEA/Gr/Al-vertical3 curve exhibits a similar pattern to the Gr/Al-vertical3 curve, with the difference that the point at which the dislocation count decreases for the second time is the same as that in the Gr/Al-vertical3 curve. In addition, the dislocation count in the HEA/Gr/Al-vertical3 model is significantly higher than that in the Gr/Al-vertical3 model before d = 32 Å.

In the curve shown in Figure 6b, the indentation force increases consistently with increasing indentation depth, which is accompanied by slight fluctuations due to the atomic characteristics of dislocations. The nucleation and reaction of dislocations releases stresses within the material, thereby reducing the applied force. As the indentation depth increases, all five models show an elastic deformation phase before transitioning to plastic deformation. Before reaching the critical point of elastic–plastic deformation, the material experiences elastic deformation without generating any dislocation defects. During this stage, the matrix deformation can be recovered elastically by the removal of the indenter.

After the critical point of elastic–plastic deformation, a notable reduction in the indentation force is observed. This decrease is attributed to the initial dislocation nucleation beneath the indenter and subsequent release of deformation energy accumulated during the elastic deformation phase of the material due to critical-point dislocation nucleation, which leads to a significant decline in the indentation force. As shown in Figure 6b, the stress values of the pure aluminum, Gr/Al-level3, Gr/Al-vertical3, HEA/Gr/Al-level3, and HEA/Gr/Al-vertical3 exhibit significant decreases at indentation depths of 9.5, 10.0, 9.2, 7.0, and 10.0 Å, respectively. Among the models, the HEA/Gr/Al-level3 model is the first to show plastic deformation, whereas the Gr/Al-level3 and HEA/Gr/Al-vertical3 models are the last to show plastic deformation. With increasing indentation depth, dislocations are expected to initiate movement, leading the matrix into the plastic deformation phase.

### 3.4. Hardness

**Figure 7:**
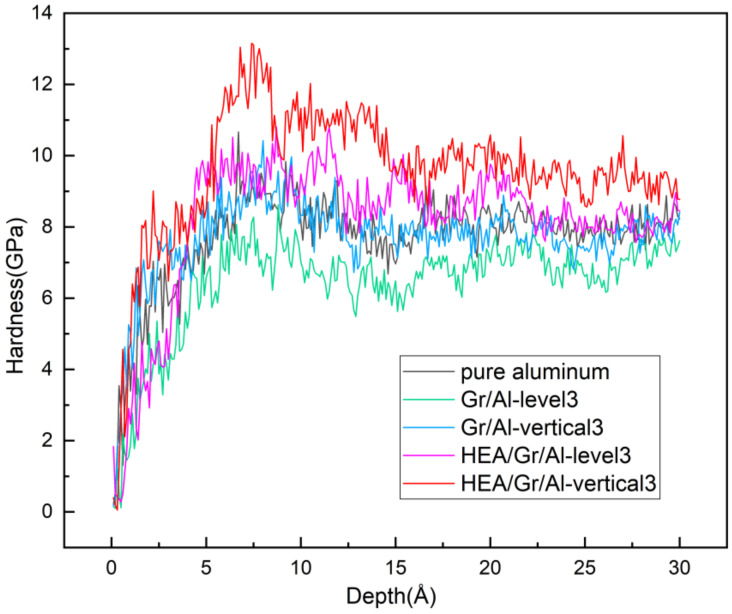
Hardness values vs. indentation depths.

Figure 7 shows the hardness vs. indentation depth curves of the five models. The relationship between hardness and indentation force is given by the following equation:

*H = p/A_c_*, (3)

where *p* represents the indentation force and *A_c_* denotes the projected contact area, which is defined as follows:

(4) $$A_{c}=\pi \left(2R-h\right)h,$$

where *R* represents the indenter radius and *h* represents the indentation depth.

As illustrated in Figure 7, the hardness values of the five models exhibit similar patterns with increasing indentation depth. Initially, the hardness gradually increases, followed by a slight decrease and eventual stabilization. However, the hardness–depth curves also exhibit a jagged shape, similar to the indentation force–displacement curves. This phenomenon is attributed to the increase in the force exerted on the workpiece with increasing indentation depth, leading to the stacking of layer dislocations and plastic deformations within the workpiece, causing a sudden decrease in hardness. In addition, the plugging of dislocations results in strain hardening, causing an increase in the hardness value with increasing indentation depth, which is consistent with the conclusions reported by Han [16]. The hardness values of the materials stabilized for indentation depths of >15 Å. The average hardness values obtained after stabilization were used as the final hardness values. The hardness values of the five models (pure aluminum, Gr/Al-level3, Gr/Al-vertical3, HEA/Gr/Al-level3, and HEA/Gr/Al-vertical3) were determined to be 8.1, 7.0, 7.8, 8.5, and 9.5 GPa, respectively. Notably, the HEA/Gr/Al-vertical3 model exhibited the highest hardness, whereas the Gr/Al-level3 model exhibited the lowest hardness. This analysis indicates that the Gr brick-and-mortar structure without the HEA coatings reduces the hardness of the Gr/Al nanocomposites. Conversely, after the coatings were introduced, the hardness values of the HEA/Gr/Al-level3 and HEA/Gr/Al-vertical3 models were 4.9% and 17.3% higher than that of pure aluminum, respectively. In addition, the brick-and-mortar structure with vertically arranged GSs effectively enhanced the hardness of the Gr/Al nanocomposites.

Among the five models, HEA/Gr/Al-vertical3 exhibited the highest dislocation density, whereas Gr/Al-level3 exhibited the lowest total dislocation density. This result aligns with the assertion made in the Taylor model [38,39] that there exists a correlation between the dislocation density and hardness within the plastic zone. The nanocomposites with GSs arranged vertically and coated with HEAs exhibit significantly higher hardness levels than other nanocomposites with equal amounts of Gr used. This can be attributed to the stiffness of GSs in the vertical orientation, providing higher support for the indenter. In addition, the incorporation of HEAs enhances the bonding between GSs and the aluminum matrix, further contributing to the overall improvement in material hardness.

The reduced hardness of the uncoated Gr/Al nanocomposites compared to that of pure aluminum can be explained by the presence of a dislocation network composed of Shockley dislocations between the indenter and GSs. This network increases the number of dislocations within the plastic zone. However, the interaction of numerous dislocations with Gr results in significant elastic deformation within the Gr facets, which reduces the contact pressure near the indenter, ultimately decreasing material hardness. These findings are consistent with those reported by Vardanyan [40].

### 3.5. Unloading Indentation Force Analysis

**Figure 8:**
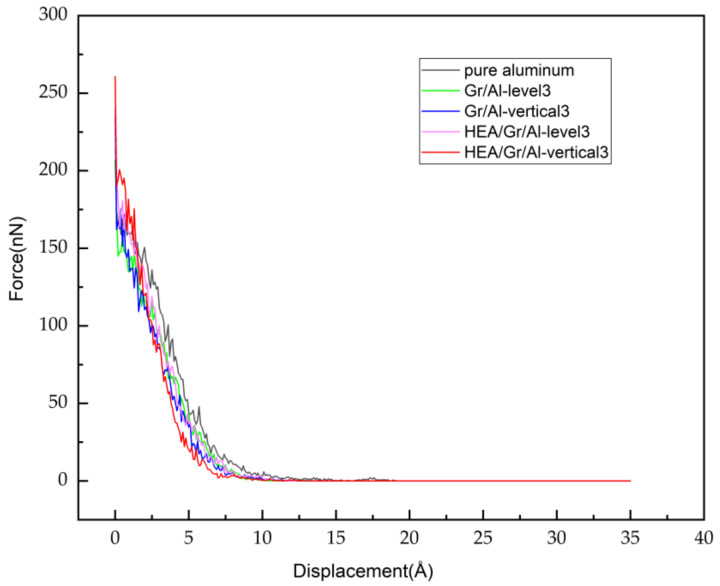
Force–displacement curves at the unloading stage.

To investigate the effect of the Gr embedding configuration on the mechanical behavior of the Gr/Al nanocomposites during the unloading stage, the relationship between the indentation force and indenter displacement for the five models was studied, as shown in Figure 8. The figure shows a gradual decrease in the indentation force from a positive magnitude with increasing indenter displacement, which is attributed to the increasing separation between the indenter and matrix and diminishing repulsive force. In the HEA/Gr/Al-level3 model, as the indenter ascends, the indentation force initially decreases to 0 at approximately 12.3 Å of the indenter displacement, while the indentation force for the pure aluminum model decreases to 0 at approximately 19 Å of the indenter displacement. This is because the distance between the indenter and surface atoms in the indentation region exceeds the range of the indentation force.

Throughout unloading, the dislocation line length decreases in all models as the indenter retracts, eventually stabilizing to a value. The total dislocation line lengths for the pure aluminum, Gr/Al-level3, Gr/Al-vertical3, HEA/Gr/Al-level3, and HEA/Gr/Al-vertical3 models were found to be 567.6, 925.1, 719.2, 533.4, and 617.4 Å, respectively. During the unloading process, the total dislocation line lengths decreased by 63.1%, 29.4%, 16.8%, 50.8%, and 47.2% for the five models, respectively. These changes in the total dislocation line length are attributed to the dislocation network evolution within the matrix induced by the indenter.

**Figure 9:**
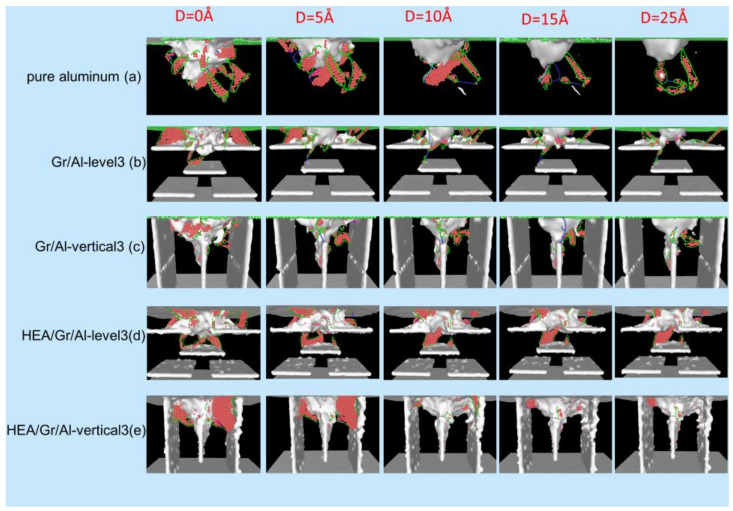
Distribution of dislocation lines and defect atoms at different indenter displacements during the unloading stage. Dislocations are colored according to their Burgers vector. Green: 1/6<112>; Dark blue: 1/2<110>; Pink: 1/6<110>; Yellow: 1/3<100>; Bright blue: 1/3<111>; Red: others. (**a**) pure aluminum; (**b**) Gr/Al-level3; (**c**) Gr/Al-vertical3; (**d**) HEA/Gr/Al-level3; (**e**) HEA/Gr/Al-vertical3.

To explore the effect of various Gr arrangements on dislocation evolution during unloading, the dislocation distributions of the five composite models were analyzed at indenter displacements of 0, 5, 10, 15, and 25 Å. These distributions are illustrated in Figure 9 using the DXA method. For the pure A1 model depicted in Figure 9a, the dislocations within the plastic zone exhibit a gradual movement in the opposite direction as the indenter is gradually lifted. The prismatic dislocation ring merges with the dislocation network beneath the indenter, leading to the gradual annihilation of dislocation defects within the matrix.

The changes in the dislocations in the composite models incorporating Gr are illustrated in Figure 9b–e. The dislocation ring moves upward along the Gr surface, merges with the dislocation network beneath the indenter, and generates a compression rod dislocation at the junction. However, as the indenter continues to rise, Shockley dislocations are consistently formed and dissolved, leading to the elimination of most dislocations within the matrix. As the indenter retracts, dislocations passing through the gap between the Gr experience stronger interactions with the Gr ends. This interaction impedes the movement of dislocations, resulting in greater dislocation retention in the Gr/Al nanocomposites. The presence of Gr acts as an obstacle to dislocation reduction during the unloading phase. In particular, the incorporation of Gr in the Gr/Al-vertical3 model exhibits the most pronounced hindering effect, whereas HEA-coated Gr exhibits a weaker hindrance in reducing the number of dislocations than uncoated Gr.

**Figure 10:**
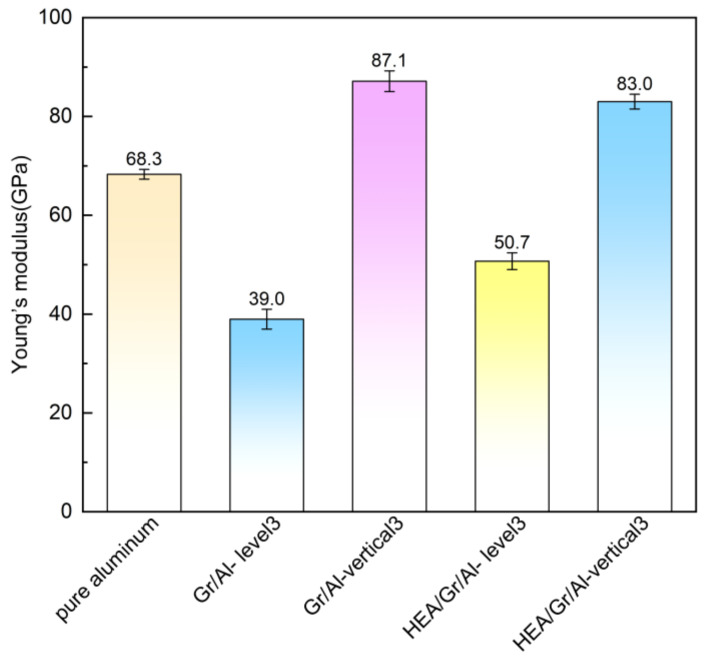
Reduced Young’s modulus of the five models.

The reduced Young’s modulus can be determined from the initial segment of the unloading force–displacement curve. This calculation is based on the formula introduced by Oliver and Pharr [41]:

(5) $$\frac{dF}{dh}=\frac{2}{\sqrt{\pi }}E_{r}\sqrt{A}$$

where dF/dh denotes the initial slope of the unloading curve, and A represents the contact area between the indenter and model at the beginning of unloading. Figure 10 shows that the Gr/Al-vertical3 model exhibits the largest Young’s modulus (87 GPa), whereas the Gr/Al-level3 model exhibits the smallest Young’s modulus (38.9 GPa). In addition, the Young’s modulus of the HEA/Gr/Al-vertical3 model is 83 GPa, which is 17.4 GPa greater than that of the pure aluminum model.

The results indicate that among the five models, the HEA/Gr/Al-vertical3 model exhibits the best mechanical properties. This finding means that when Gr is arranged vertically in a brick-and-mortar structure in a Gr/Al composite, the elasticity modulus and hardness of the composite increase by 21.5% and 17.3%, respectively, compared with those of pure aluminum.

### 3.6. Analysis of Dislocation Types

**Figure 11:**
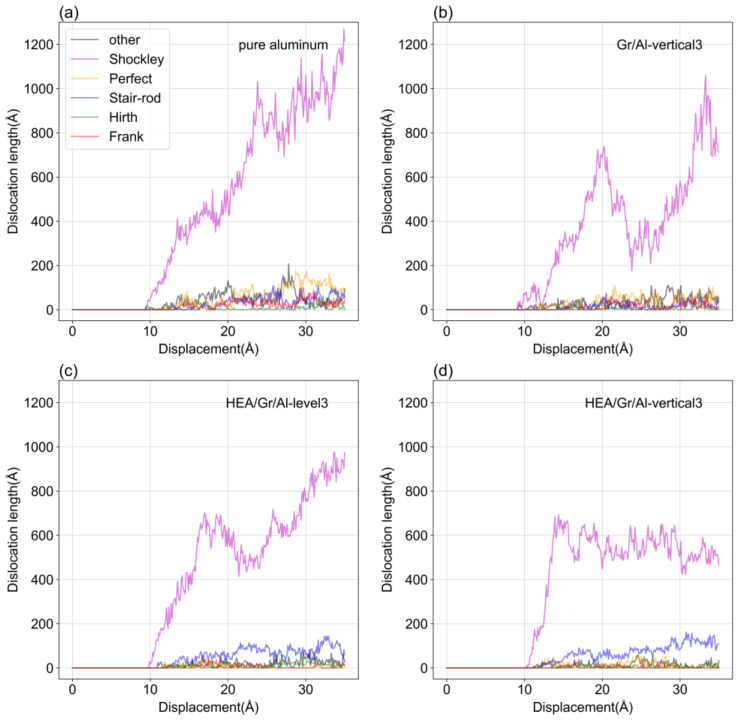
Dislocation length–indenter displacement curves. (**a**) pure aluminum; (**b**) Gr/Al-vertical3; (**c**) HEA/Gr/Al-level3; (**d**) HEA/Gr/Al-vertical3.

This study specifically analyzes the different types of dislocations present in the loading process of three models and a pure aluminum model. Figure 11 illustrates that Shockley dislocations dominate the dislocations generated during the loading process of the four models. In Figure 11a, the Shockley dislocations in the pure aluminum model exhibit a consistent upward trend, reaching a final value of 1216 Å, whereas the number of Hirth dislocations is nearly negligible. Conversely, the numbers of Other, Perfect, and Frank dislocations fluctuate around certain values, which are higher than those observed in the other three models. These observations suggest that the presence of grain boundaries is harmful to the nucleation of these three types of dislocations.

In Figure 11b–d, the number of Shockley dislocations exhibits significant fluctuations, which are primarily attributed to the presence of GSs impeding dislocation growth. In addition, Figure 11c,d show that the number of Stair-rod dislocations in the HEA/Gr/Al-level3 and HEA/Gr/Al-vertical3 models exceeds that of the other two models. Conversely, the number of Other, Perfect, and Frank dislocations is lower than that in the other two models, probably due to the integration of HEA coatings, which promote the initiation of Stair-rod dislocations.

## 4. Conclusions

In this study, two arrangements of the brick-and-mortar structure were introduced in Gr/Al nanocomposites, and corresponding models were developed with and without HEA coatings. A comprehensive comparison was performed on the evolution of dislocations and variations in the indentation force, hardness, and elastic modulus of the nanocomposites during nanoindentation testing between the pure aluminum and Gr/Al nanocomposite models. In addition, detailed analyses of the dislocation network generated by indentation, mechanism of flake interactions, and their effects on material hardness were performed. The following results were obtained:

(1) Dislocation growth was significantly hindered by GSs, and the dislocation network generated by the indenter in the plastic zone around it could not penetrate the Gr layer.
(2) The interactions between dislocations and GSs in the Gr/Al nanocomposites, where Gr was structured in a brick-and-mortar configuration, induced significant elastic deformation within the Gr layers. This deformation reduced the contact pressure near the indenter, resulting in decreased hardness of the nanocomposites compared with that of pure aluminum.
(3) The vertical arrangement of uncoated Gr with a brick-and-mortar structure effectively increased the elastic modulus of the Gr/Al nanocomposites by approximately 27.7%.
(4) The incorporation of HEA coatings on Gr significantly enhanced the hardness of the Gr/Al nanocomposites, which was most pronounced when Gr was arranged vertically with a brick-and-mortar structure, exhibiting elastic modulus and hardness values of 83 and 9.5 GPa, respectively. These values were improved by 21.5% and 17.3%, respectively, compared with those of pure aluminum.

MD simulations of the nanoindentation testing of the Gr/Al nanocomposites coated with HEAs revealed that the composite with an HEA mass fraction of 3.4% and vertically aligned GSs showed significantly enhanced mechanical properties. This finding not only provides valuable insights into Gr/Al nanocomposites but also establishes a foundation for future research on the effects of various factors, such as HEA content and dislocation type, on the mechanical properties of such nanocomposites. Owing to the limited experimental methods currently available to validate the conclusions of this study, we anticipate that future technological advancements will facilitate the development of more reliable experimental techniques capable of corroborating the results obtained from MD simulations.

## Figures and Tables

**Table 1:** Potential parameters for metal–metal interactions.

Atom	*ε* (eV)	*σ* (nm)
Al-C	0.035078	0.30135
Cr-C	0.0348	0.28182
Co-C	0.0032	0.14091
Fe-C	0.0356	0.28092
Ni-C	0.0354	0.27855

## Data Availability

The original contributions presented in the study are included in the article, further inquiries can be directed to the corresponding author.
